# Diagnóstico Errôneo de Angiossarcoma Cardíaco na Era COVID-19

**DOI:** 10.36660/abc.20220501

**Published:** 2023-07-27

**Authors:** Arash Amin, Zohreh Taheri, Mahshid Hesami, Azin Alizahehasl, Zeinab Norouzi, Nasibeh Mohammadi

**Affiliations:** 1 Lorestan Heart Center Lorestan University of Medical Sciences Khorram-abad Irã Lorestan Heart Center (Madani hospital), Lorestan University of Medical Sciences (LUMS), Khorram-abad – Irã; 2 Cardio-Oncology Research Center Rajaie Cardiovascular, Medical & Research Center Iran University of Medical Science Tehran Irã Cardio-Oncology Research Center, Rajaie Cardiovascular, Medical & Research Center, Iran University of Medical Science (IUMS), Tehran – Irã; 3 Mousavi Hospital Zanjan University of Medical Sciences Zanjan Irã Mousavi Hospital, Zanjan University of Medical Sciences (ZUMS), Zanjan – Irã

**Keywords:** Erro de Diagnóstico, Angiosarcoma/cirurgia, Neoplasias Cardíacas, COVID-19/infecção, Imagem Multimodal/métodos, Diagnóstico por Imagem/métodos

## Abstract

Os últimos meses de 2019 foram marcados pelo surgimento de uma nova pandemia, denominada “COVID-19”. Desde então, essa infecção e suas complicações têm sido a prioridade de profissionais de saúde, com muitos sintomas atribuídos às suas apresentações precoces e tardias. Até o momento, outras doenças, mesmo em situações fatais, têm sido negligenciadas ou diagnosticadas incorretamente devido à atribuição dos sintomas do paciente à presença da infecção por COVID-19. Apresentamos aqui um caso de angiossarcoma cardíaco, em um menino que, cerca de 2 meses antes, havia sido infectado com COVID-19. Dado o histórico de infecção, a abordagem inicial foi o manejo da miopericardite pós-COVID-19. No entanto, o quadro do paciente piorou, exigindo reavaliação por multimodalidades com maior precisão. Por fim, o paciente foi diagnosticado com um tumor cardíaco. Este artigo procura enfatizar a importância da atenção a outras doenças e condições fatais na era COVID-19, com ênfase em evitar diagnósticos incorretos de outras doenças.

## Introdução

Aproximadamente 90% dos tumores cardíacos são benignos e apenas alguns são malignos.^1^ A maioria dos tumores cardíacos malignos são sarcomas, incluindo o angiossarcoma.^2^ O angiossarcoma pode estar presente em qualquer idade, embora os pacientes geralmente tenham entre 40 e 50 anos.^3^ Este tumor, cuja origem é comumente o átrio direito (AD),^4^ pode ser totalmente assintomático, sendo frequentemente detectado de forma não intencional. Além disso, os pacientes podem apresentar dispneia, desconforto torácico, arritmia, derrame pericárdico (DP), tamponamento, insuficiência cardíaca (IC), pré-síncope ou síncope, sintomas constitucionais e eventos tromboembólicos. Um ecocardiograma mostrou uma massa heterogênea com áreas hemorrágicas ou necróticas. O prognóstico do angiossarcoma primário é ruim, mesmo com ressecção cirúrgica completa.^5^

Um novo coronavírus chamado “COVID-19” tem se alastrado pelo mundo desde o final de 2019 e início de 2020.^6^ Até o momento, outras doenças, mesmo em situações fatais, têm sido negligenciadas ou diagnosticadas incorretamente devido à atribuição dos sintomas do paciente à presença da infecção por COVID-19. Este artigo apresenta um caso de angiossarcoma cardíaco em um menino que recebeu um diagnóstico inadequado de complicação dessa infecção.

### Relato de Caso

Um menino de 17 anos com histórico de infecção por COVID-19 dois meses antes apresentou palpitações paroxísticas, dispneia e fadiga que duraram duas semanas. Além disso, paciente referia dor torácica pleurítica localizada em hemitórax direito e hiperidrose.

**Figura 1:**
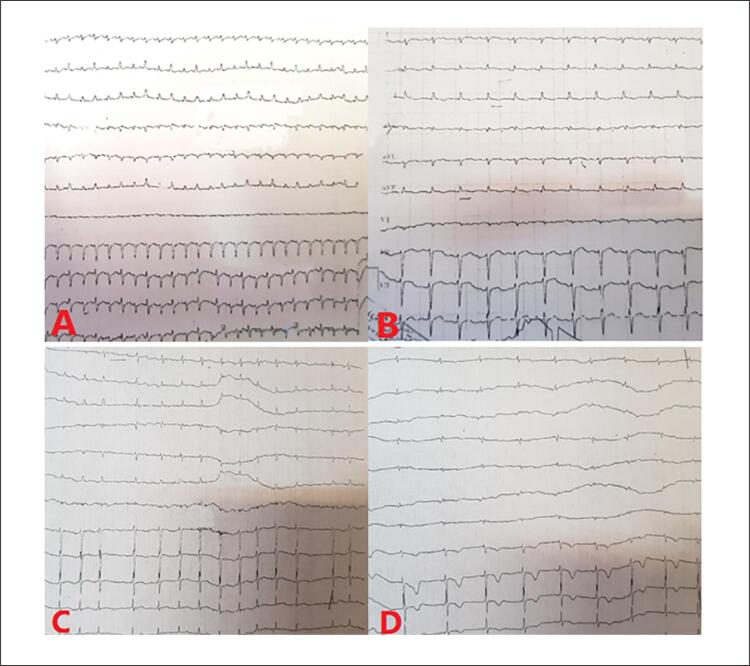
– As imagens mostram em A) complexos QRS de baixa tensão e TVPS com alternância elétrica, B) taquicardia sinusal com supradesnível ST inferior, C) ritmo de FA e D) complexos QRS de baixa tensão, ritmo atrial baixo (bradicardia) e alterações significativas de ST-T em V1-V3. TPSV: taquicardia supraventricular paroxística; FA: fibrilação atrial.

Ao exame físico, o paciente apresentava aparência geral pálida, com pressão arterial de 95/65 mmHg, frequência cardíaca de 190 batimentos por minuto, frequência respiratória de 24 respirações por minuto e temperatura corporal de 38,6 °C. Um eletrocardiograma (ECG) revelou taquicardia paroxística supraventricular (TPSV) ( Figura 1A ), posteriormente tratada com injeção de adenosina. Exames laboratoriais demonstraram anemia, leucocitose e níveis elevados de reagentes de fase aguda ( Tabela 1 ). O ecocardiograma transtorácico (ETT) mostrou fração de ejeção do ventrículo esquerdo (FEVE) de 55%, pressão arterial pulmonar de 40 mmHg e DP leve a moderado.

**Tabela 1:** – Resultados dos exames laboratoriais iniciais do paciente

21900 cmm: WBC
70%: PMN
Linf.: 5%
Banda: 25%
AST: 18 U/L
ALT: 20 U/L
ALP: 478 U/L
T Bil: 0,6 µmol/L
D Bil: 0,24 µmol/L
Hb: 9,9 g/dl
Hct: : 33,1 L/L
RBC: 4130 milhões/mm^3^
VCM: 80,1µm^3^
HCM: 24 pg/célula
CHCM: 29,9 pg/célula
B/C x 2: Negativo
U/A: Normal
U/C: Negativo
Plt: 54500/µl
Troponina: 0,03 ng/ml
Ferritina: 889 µg/l
VHS: 74 mm/h
+: PCR

WBC: leucócitos; PMN: polimorfonuclear; Linf.: linfócitos; Banda: neutrófilos de banda; Hb: hemoglobina; Hct: hematócrito; RBC: hemácias; VCM: volume corpuscular médio; HCM: hemoglobina corpuscular média; CHCM: concentração média de hemoglobina corpuscular; Plt: teste de contagem de plaquetas; AST: aspartato aminotransferase; ALT: alanina transaminase; ALP: fosfatase alcalina; T Bil: bilirrubina total; D Bil: bilirrubina direta; B/C: hemocultura; U/A: urinálise; U/C: cultura de urina; VHS: velocidade de hemossedimentação; PCR: proteína C reativa; cmm: células por milímetro cúbico; g/dl: gramas por decilitro; L/L: litro de células por litro de sangue; milhão/mm3: milhões por milímetro cúbico; μm3: mícrons cúbicos; pg/célula: picogramas por célula; μl: microlitro; U/L: unidades por litro; µmol/L: micromol por litro; ng/ml: nanogramas por mililitro; μg/l: microgramas por litro; mm/h: milímetros por hora.

**Figura 2:**
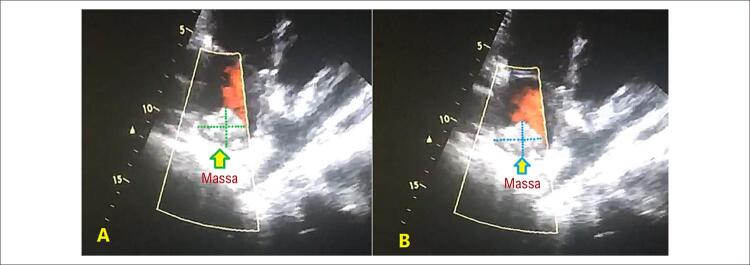
– ETT por Doppler colorido, evidenciando uma massa extracardíaca invadindo o AD e exercendo efeitos compressivos sobre a parte distal da VCS. ETT: ecocardiograma transtorácico; AD: átrio direito; VCS: veia cava superior.

**Figura 3:**
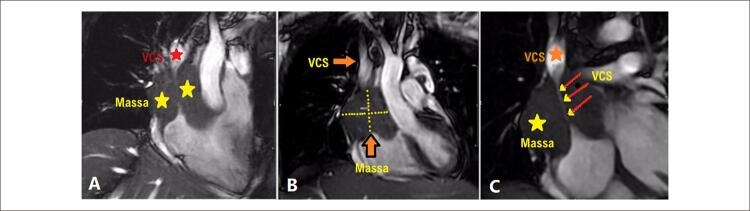
– As imagens A) e B) mostram uma massa intrapericárdica circundando a parte distal da VCS, com invasão mínima da massa até a borda superior do AD, e a imagem C) mostra o tumor e o estreitamento acentuado da VCS. VCS: veia cava superior; AD: átrio direito.

**Figura 4:**
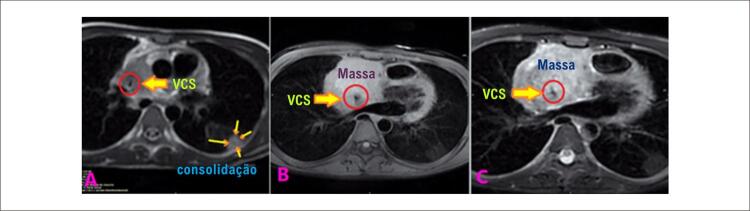
– A imagem A) mostra a ressonância magnética cardiovascular com consolidação de base pleural no lobo superior do pulmão esquerdo e estreitamento acentuado da VCS, e as imagens B) e C) ilustram o tumor e o estreitamento acentuado da VCS. VCS: veia cava superior.

**Figura 5:**
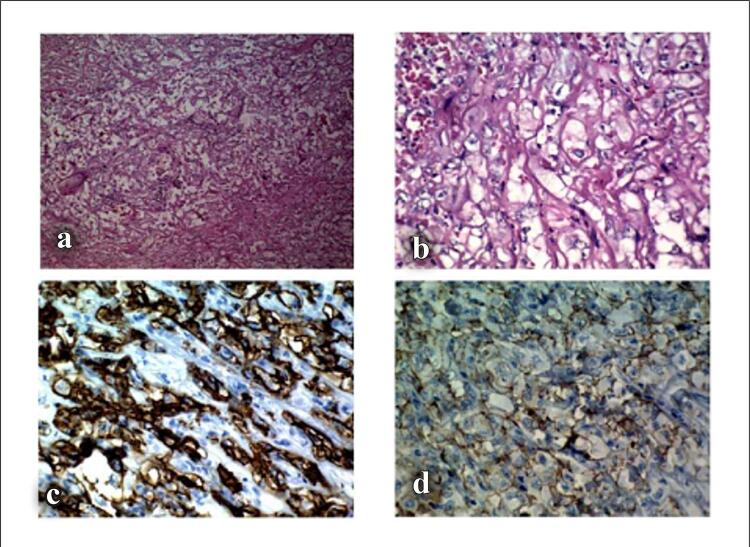
– O exame microscópico (a e b) mostra espaços vasculares arborizados revestidos por células epitelioides pleomórficas e múltiplos espaços capilares com células endoteliais atípicas. Espaços vasculares anastomosados, extravasamento de hemácias, necrose maciça e mitoses frequentes estão presentes. Na coloração imunohistoquímica, as células tumorais foram positivas para CD34(c) e CD31(d) e negativas para CK, WT1, Calretinina e D2-4 (não mostrado).

**Figura 6:**
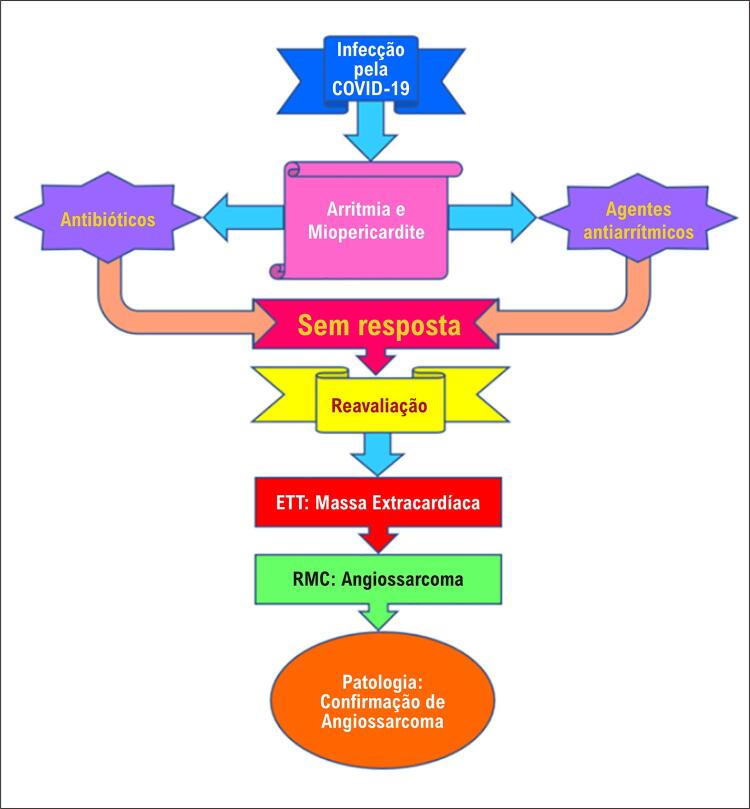
– A linha do tempo da apresentação e tratamento do paciente. ETT: ecocardiograma transtorácico; RMC: ressonância magnética cardiovascular.

O paciente foi admitido com o diagnóstico inicial de miopericardite devido a uma infecção por COVID-19. Durante a internação, sua arritmia refratária ( Figura 1: B , C e D ) induziram a administração de outras medicações, incluindo verapamil. Uma avaliação mais aprofundada indicou anemia persistente e leucocitose exacerbada em medições seriadas. A contagem inicial de leucócitos (WBC) foi de 21.900 células/mm^3^, aumentando para 38.800 células/mm^3^ durante a internação. Outro ETT revelou massa extracardíaca invadindo o AD ( Figura 2 ). Posteriormente, uma ressonância magnética cardiovascular (RMC) mostrou uma grande massa pericárdica com diâmetro máximo de 77 × 62 mm ao redor da parte distal da veia cava superior (VCS). A RMC também evidenciou o estreitamento da VCS pelo efeito compressivo do tumor ( Figura 3 ) e uma consolidação pleural de 21,7 × 13,3 mm no lobo superior do pulmão esquerdo ( Figura 4 ). A massa apresentava-se homogeneamente isointensa em relação ao miocárdio normal, altamente suspeita de angiossarcoma. O paciente foi encaminhado para nova avaliação. Uma vez confirmado o diagnóstico, o plano de tratamento foi definido. Por fim, o paciente foi submetido a uma cirurgia cardíaca e o tumor foi ressecado. O estudo anatomopatológico confirmou o diagnóstico ( Figura 5 ), e um plano de tratamento adicional e acompanhamento foram considerados ( Figura 6 ).

## Discussão

O angiossarcoma cardíaco primário constitui o tipo mais comum de tumor cardíaco maligno.^6^ Os homens têm maior probabilidade de desenvolver esse tipo de tumor,^7^ especialmente na faixa dos 50 anos.^3^ Nosso paciente era um menino de 17 anos, sendo muito mais jovem do que a idade esperada.

O padrão do angiossarcoma cardíaco primário pode ser esporádico ou familiar, sendo a taxa de sobrevida significativamente menor em pacientes com o padrão familiar.^8^ Nosso paciente não tinha histórico de avaliação genética, nem relatou história familiar de doença semelhante. Recomendamos que a família imediata desse grupo de pacientes seja submetida a avaliação médica e genética.

O sítio mais comum de angiossarcoma é o AD.^4^ Hirai et al.,^9^ relataram um caso de oclusão da artéria coronária pelo efeito compressivo de um angiossarcoma cardíaco, que trataram com sucesso por meio de intervenção coronária percutânea (PCI). O angiossarcoma de nosso paciente se manifestou como uma massa extracardíaca, invadindo o AD e exercendo efeito de compressão sobre a parte distal da VCS, resultando em estreitamento severo deste vaso.

Pacientes com angiossarcoma podem apresentar dispneia, desconforto torácico, DP, tamponamento, IC, pré-síncope, síncope, sintomas constitucionais e eventos tromboembólicos.^5^ Os sintomas iniciais do nosso paciente foram fadiga, hiperidrose, palpitações paroxísticas e dor torácica pleurítica no hemitórax direito. Cerqueira et al,.^10^ descreveram um caso de angiossarcoma volumoso em ventrículo direito (VD) com apresentação inicial de hiporexia e IC.

O sistema condutivo pode ser lesado pela infiltração tumoral, gerando taquiarritmia ou bradiarritmia.^11^ O primeiro caso de angiossarcoma cardíaco com apresentação inicial de taquicardia atrial incessante foi relatado por Nguyen et al.,^12^ em 2021. Em nosso paciente, a invasão tumoral do sistema condutivo pode ter levado à TPSV refratária, taquicardia sinusal e fibrilação atrial. Até onde sabemos, essa apresentação com diferentes arritmias não é frequente em pacientes com angiossarcoma.

Em pacientes com angiossarcoma, os sinais vitais e mesmo o ECG podem se apresentar como normais; no entanto, radiografias de tórax (RXT) e ecocardiogramas podem visualizar DP e abaulamento tumoral.^13^ Luo et al.,^14^ relataram um caso de angiossarcoma no apêndice atrial direito, diagnosticado por ecocardiograma transesofágico. O DP do nosso paciente foi diagnosticado no primeiro ETT, mas o tumor não foi localizado. Diagnosticar angiossarcoma apenas por ETT não é fácil; portanto, é essencial focar em todas as câmaras cardíacas.

Nosso paciente tinha um histórico recente de infecção por COVID-19 e estava em estado crítico. Consequentemente, o foco inicial foi o diagnóstico das possíveis complicações da infecção, o que levou à desatenção a outros aspectos do ETT após a observação do DP. A ineficácia do tratamento do paciente e a exacerbação subsequente de sua condição exigiram uma reavaliação. Por fim, no segundo ETT realizado por outro operador, a massa foi identificada.

A tomografia computadorizada (TC) pode conferir dados adicionais sobre o tumor e desempenhar um papel significativo no diagnóstico de metástases.^15^ A RMC é mais valiosa para estudar anormalidades dos tecidos moles e descartar outras patologias, incluindo trombose.^16^ Em nosso caso, a RMC ajudou a confirmar a diagnóstico descrevendo as características do tumor e sua metástase para o lobo superior do pulmão esquerdo. Os pulmões são o órgão mais comumente metastatizado pelo angiossarcoma. Além disso, o envolvimento tumoral pode ocorrer em outros sítios, como linfonodos, fígado, ossos, baço e glândulas adrenais.^17^

Nosso caso mostra que o diagnóstico de uma suspeita de angiossarcoma requer um ecocardiograma, além de uma anamnese abrangente. No entanto, a RXT, a TC e a RMC podem ajudar a estabelecer um diagnóstico preciso. Arktout et al.,^18^ relataram um caso de malignidade cardíaca em um homem jovem com dor torácica no hemitórax direito e enfatizaram a importância da imagem multimodal no diagnóstico de tais tumores.

O primeiro passo no diagnóstico e tratamento de pacientes jovens com sintomas cardíacos na era COVID-19 deve ser a atenção adequada aos sintomas e a anamnese detalhada. A dispneia persistente e o desconforto torácico sem origem específica em pacientes jovens podem nos levar ao diagnóstico de tumor.^19^ No entanto, tumores cardíacos também devem ser considerados em pacientes idosos. Linfeng et al.,^20^ descreveram um homem de 65 anos com quadro de dispneia indeterminada, desconforto torácico, tontura e fraqueza em membros inferiores, finalmente diagnosticado como angiossarcoma.

Nosso paciente foi inicialmente diagnosticado erroneamente devido à anamnese incompleta e uso inadequado das modalidades de imagem disponíveis na primeira avaliação. Sua história recente de infecção por COVID-19 levou ao primeiro diagnóstico de miopericardite pós-COVID-19. No entanto, sua condição de piora exigiu avaliação adicional por RMC e ETT, o que ajudou a estabelecer o diagnóstico adequado. Na era COVID-19, outras doenças, e até mesmo condições com risco à vida, como doenças cardiovasculares, parecem ter sido ignoradas ou subestimadas.

Para concluirmos, a infeção por COVID-19 não deve ser motivo para diagnósticos incorretos ou adiamento de processos diagnósticos e terapêuticos relativos a outras doenças. Na realidade, as abordagens consagradas devem ser continuadas, embora com a devida atenção aos protocolos de proteção do COVID-19.
