# Supplementary material for: Diagnóstico Errôneo de Angiossarcoma Cardíaco na Era COVID-19
Source: Arq Bras Cardiol. 2023 Jul 27;120(7):e20220501. [Article in Portuguese] doi: 10.36660/abc.20220501 (PMC10382138; doi:10.36660/abc.20220501)
Supplement: Translation

# Translation: Misdiagnosis of a Cardiac Angiosarcoma During the COVID-19 Pandemic

**Authors:** Arash Amin, Zohreh Taheri, Mahshid Hesami, Azin Alizahehasl, Zeinab Norouzi, Nasibeh Mohammadi

**Affiliations:** 1 Iran Lorestan Heart Center (Madani hospital), Lorestan University of Medical Sciences (LUMS), Khorram-abad – Iran; 2 Iran Cardio-Oncology Research Center, Rajaie Cardiovascular, Medical & Research Center, Iran University of Medical Science (IUMS), Tehran – Iran; 3 Iran Mousavi Hospital, Zanjan University of Medical Sciences (ZUMS), Zanjan – Iran

**Keywords:** Diagnostic Errors, Angiosarcoma/surgery, Heart Neoplasms, COVID-19/infection, Multimodal Imaging/diagnostic, Imaging/methods

## Abstract

The final months of 2019 saw the emergence of a new pandemic termed “COVID-19”. Since then, this infection and its complications have been the priority of healthcare providers, with many symptoms attributed to its early and late presentations. Thus far, other diseases, even fatal situations, have been overlooked or misdiagnosed due to the attribution of patient symptoms to the presence of COVID-19 infection. We herein present a case of cardiac angiosarcoma in a young boy who had previously become infected with COVID-19 about two months earlier. Given the history of infection, the initial approach was post-COVID-19 myopericarditis management. However, the patient’s condition worsened, necessitating reevaluation via multimodalities with higher precision. Ultimately, the patient was diagnosed with a cardiac tumor. This article seeks to underscore the significance of taking heed of other diseases and fatal conditions during the COVID-19 pandemic with an emphasis on avoiding misdiagnosing other diseases.

## Introduction

Approximately 90% of cardiac tumors are benign, and only a few are malignant.^1^ Most malignant heart tumors are sarcomas, including angiosarcoma.^2^ Angiosarcoma can be present at any age, although patients are usually between 40 and 50 years old.^3^ This tumor, whose origin is commonly the right atrium (RA),^4^ may be completely asymptomatic and is often detected unintentionally. In addition, patients may have dyspnea, chest discomfort, arrhythmia, pericardial effusion (PE), tamponade, heart failure (HF), presyncope or syncope, constitutional symptoms, and thromboembolic events. An echocardiogram showed a heterogeneous mass with hemorrhagic or necrotic areas. The prognosis of primary angiosarcoma is poor, even with complete surgical resection.^5^

A new coronavirus called “COVID-19” has been sweeping the world since the end of 2019 and the beginning of 2020.^6^ Thus far, other diseases, even fatal situations, have been overlooked or misdiagnosed due to the attribution of patient symptoms to the presence of COVID-19 infection. This article presents a case of cardiac angiosarcoma in a young boy misdiagnosed as a complication of this infection.

### Case Report

A 17-year-old boy with a history of COVID-19 infection two months earlier presented with paroxysmal palpitations, dyspnea, and fatigue that lasted two weeks. The patient also mentioned localized pleuritic chest pain in the right hemithorax and hyperhidrosis.

On physical examination, the patient was generally pale and had a blood pressure of 95/65 mmHg, a pulse rate of 190 beats per minute, a respiratory rate of 24 breaths per minute, and a body temperature of 38.6 °C. An electrocardiogram (ECG) revealed paroxysmal supraventricular tachycardia (PSVT) ( Figure 1 A ), subsequently managed with adenosine injection. Laboratory tests demonstrated anemia, leukocytosis, and elevated levels of acute-phase reactants ( Table 1 ). Transthoracic echocardiogram (TTE) showed a left ventricular ejection fraction (LVEF) of 55%, a pulmonary artery pressure of 40 mmHg, and mild-to-moderate PE.

**Figure 1:** – Images show in A) low-voltage QRS complexes and PSVT with electrical alternans, B) sinus tachycardia with inferior ST elevation, C) AF rhythm, and D) low-voltage QRS complexes, low atrial rhythm (bradycardia), and significant ST-T changes in V1-V3. PSVT: paroxysmal supraventricular tachycardia; AF: atrial fibrillation.

**Table 1:** – The initial laboratory test results of the patient

21900 cmm: WBC
70%: PMN
Lymph.: 5%
Band: 25%
AST: 18 U/L
ALT: 20 U/L
ALP: 478 U/L
T Bil: 0,6 µmol/L
D Bil: 0,24 µmol/L
Hb: 9,9 g/dl
Hct: 33,1 L/L
RBC: 4130 million/mm^3^
MCV: 80.1µm^3^
MCH: 24 pg/cell
MCHC: 29.9 pg/cell
B/C x 2: Negative
U/A: Normal
U/C: Negative
Plt: 54500/µl
Troponin: 0.03 ng/ml
Ferritin: 889 µg/l
ESR: 74 mm/h
+: CRP

WBC: white blood cells; PMN: polymorphonuclear; Lymph: lymphocytes; Band: band cells; Hb: hemoglobina; Hct: hematocrit; RBC: red blood cells; MCV: mean corpuscular volume; MCH: mean corpuscular hemoglobina; MCHC: mean corpuscular hemoglobin concentration; Plt: platelet count test; AST: aspartate aminotransferase; ALT: alanine transaminase; ALP: alkaline phosphatase; T Bil: total bilirubin; D Bil: direct bilirubin; B/C: blood culture; U/A: urinalysis; U/C: urine culture; ESR: erythrocyte sedimentation rate; CRP: c-reactive protein; cmm: cells per cubic millimeter; g/dl: grams per decilitre; L/L: liter of cells per liter of blood; million/mm3: millions per cubic millimeter; μm3: cubic microns; pg/cell: picograms per cell; μl: microliter; U/L: units per liter; µmol/L: micromole per liter; ng/ml: nanograms per milliliter; μg/l: micrograms per liter; mm/h: millimeters per hour.

The patient was admitted with the initial diagnosis of myopericarditis due to a COVID-19 infection. During the hospitalization, his refractory arrhythmia ( Figure 1 B , C , and D ) prompted the administration of other medications, including verapamil. Further evaluation indicated persistent anemia and exacerbated leukocytosis in serial measurements. The initial white blood cell count (WBC) was 21,900 cells/mm^3^, which rose to 38,800 cells/mm^3^during the hospital stay. Another TTE examination revealed an extracardiac mass invading the RA ( Figure 2 ). Afterward, cardiovascular magnetic resonance (CMR) imaging showed a large pericardial mass with a maximum diameter of 77 × 62 mm surrounding the distal part of the superior vena cava (SVC). CMR also visualized the narrowing of the SVC by the compression effect of the tumor ( Figure 3 ) and a 21.7 × 13.3 mm pleural-based consolidation in the upper lobe of the left lung ( Figure 4 ). The mass appeared homogeneously isointense relative to the normal myocardium, highly suspicious of an angiosarcoma. He was referred for further evaluation. Once the diagnosis was confirmed, a treatment plan was devised. Finally, the patient underwent cardiac surgery, and the tumor was resected. The pathological study confirmed the diagnosis ( Figure 5 ), and further treatment planning and follow-up planning were considered ( Figure 6 ).

**Figure 2:** – TTE by Color Doppler study reveals an extracardiac mass invading the RA and exerting compression effects on the distal part of the SVC. TTE: transthoracic echocardiogram; RA: right atrium; SVC: superior vena cava.

**Figure 3:** – Images A) and B) show an intrapericardial mass surrounding the distal part of the SVC, with minimal invasion of the mass to the upper border of the RA, and image C) depicts the tumor and the severe narrowing of the SVC. SVC: superior vena cava; RA: right atrium.

**Figure 4:** – Image A) shows cardiovascular magnetic resonance imaging with a pleural-based consolidation in the left lung’s upper lobe and the SVC’s severe narrowing, and images B) and C) illustrate the tumor and the severe narrowing of the SVC. SVC: superior vena cava.

**Figure 5:** – Microscopic examination (a and b) shows arborizing vascular spaces lined by pleomorphic epithelioid cells and many capillary-like vascular spaces with atypical endothelial cells. Anastomosing vascular spaces, RBC extravasation, massive necrosis and frequent mitoses are present. On Immunohistochemical staining tumoral cells show positivity for CD34(c) and CD31(d) and negative for CK, WT1, Calretinin and D2- 4 (not shown).

**Figure 6:** – The timeline of the patient’s presentation and management. TTE: transthoracic echocardiogram; CMR: cardiovascular magnetic resonance.

## Discussion

Primary cardiac angiosarcoma constitutes the most common type of malignant cardiac tumor.^6^ Males are more likely to develop this tumor,^7^ especially in their 50s.^3^ Our patient was a 17-year-old boy, much younger than the expected age.

The pattern of primary cardiac angiosarcoma may be sporadic or familial, with the survival rate being significantly lower in patients with the latter.^8^ Our patient had no history of genetic evaluation, nor did he report a family history of a similar disease. We recommend that the immediate family of this group of patients undergo genetic and medical assessments.

The most common site of angiosarcoma is the RA.^4^ Hirai et al.^9^ reported a case of coronary artery occlusion due to the compression effect of a cardiac angiosarcoma, which they successfully treated via percutaneous coronary intervention (PCI). Our patient’s angiosarcoma manifested itself as an extracardiac mass invading the RA and exerting compression effects on the distal part of the SVC, resulting in its severe narrowing.

Patients with angiosarcoma may present dyspnea, chest discomfort, PE, tamponade, HF, presyncope, syncope, constitutional symptoms, and thromboembolic events.^5^ Our patient’s initial symptoms were fatigue, hyperhidrosis, paroxysmal palpitations, and pleuritic chest pain in the right hemithorax. Cerqueira et al.^10^ described a case of a massive angiosarcoma in the right ventricle (RV) with the initial presentation of appetite loss and HF.

The conductive system may be injured by tumor infiltration, generating tachyarrhythmia or bradyarrhythmia.^11^ The first case of cardiac angiosarcoma with the initial presentation of incessant atrial tachycardia was reported by Nguyen et al.^12^ in 2021. In our patient, tumor invasion of the conductive system might have produced his refractory PSVT, sinus tachycardia, and atrial fibrillation. To our knowledge, this presentation with different arrhythmias is not frequent in patients with angiosarcoma.

In patients with angiosarcoma, vital signs or even ECG may become normal; nevertheless, chest X-rays (CXR) and echocardiograms can visualize PE and tumor bulging.^13^ Luo et al.^14^ reported a case of angiosarcoma in the RA appendage, diagnosed via transesophageal echocardiogram. Our patient’s PE was diagnosed on the first TTE, but the tumor was missed. Diagnosing angiosarcoma via TTE alone is not easy; it is, therefore, essential to focus on all cardiac chambers.

Our patient had a recent history of COVID-19 infection and was in critical condition. Consequently, the initial focus was on diagnosing the possible complications of the infection, which led to inattention to other aspects of the TTE examination after it revealed PE. The ineffectiveness of the patient’s treatment and the next exacerbation of his condition necessitated reevaluation. Finally, on the second TTE examination performed by another operator, the mass was diagnosed.

Computerized tomography (CT) scans can confer additional data regarding the tumor and play a significant role in diagnosing metastasis.^15^ CMR is more valuable for studying soft tissue abnormalities and ruling out other pathologies, including thrombosis.^16^ In our case, CMR helped confirm the diagnosis by depicting the features of the tumor and its metastasis to the upper lobe of the left lung. Lungs are the most common organ metastasized by angiosarcoma. Still, tumor involvement may occur in other sites, such as lymph nodes, liver, bone, spleen, and adrenal glands.^17^

Our case shows that the diagnosis of a suspected angiosarcoma needs an echocardiogram in addition to comprehensive history taking. Nonetheless, CXR, CT, and CMR can help establish a precise diagnosis. Arktout et al.^18^ reported a case of cardiac malignancy in a young man with chest pain in the right hemithorax and emphasized the significance of multimodal imaging in diagnosing such tumors.

The first step in diagnosing and managing young patients with cardiac symptoms during the COVID-19 pandemic should be adequate attention to symptoms and detailed history taking. Persistent dyspnea and chest discomfort in young patients without a specific source can lead us to the tumor diagnosis.^19^ However, cardiac tumors should be considered in elderly patients, too. Linfeng et al.^20^ described a 65-year-old man with undetermined dyspnea, chest discomfort, dizziness, and weakness of the lower limbs, finally diagnosed as angiosarcoma.

Our patient was initially misdiagnosed due to incomplete history taking and improper use of available imaging modalities in the first evaluation. His recent history of COVID-19 infection led to the first diagnosis of post-COVID-19 myopericarditis. Nevertheless, his worsening condition mandated further evaluation via CMR and TTE, which helped establish the appropriate diagnosis. It seems that during the COVID-19 pandemic, other diseases, even life-threatening conditions such as cardiovascular diseases, have been ignored or postponed.

In conclusion, COVID-19 infection should not be a reason for the misdiagnosis or postponement of diagnostic and therapeutic processes concerning other diseases. Indeed, time-honored approaches to patients should be continued, albeit with due attention to COVID-19 protective protocols.
